# Rat microbial biogeography and age-dependent lactic acid bacteria in healthy lungs

**DOI:** 10.1038/s41684-023-01322-x

**Published:** 2024-01-31

**Authors:** Lan Zhao, Christine M. Cunningham, Adam M. Andruska, Katharina Schimmel, Md Khadem Ali, Dongeon Kim, Shenbiao Gu, Jason L. Chang, Edda Spiekerkoetter, Mark R. Nicolls

**Affiliations:** 1 Department of Medicine, Division of Pulmonary, Allergy, and Critical Care Medicine, Stanford, CA USA; 2 VA Palo Alto Health Care System, Palo Alto, CA USA; 3 Vera Moulton Wall Center for Pulmonary Vascular Disease, Stanford, CA USA

**Keywords:** Microbiome, Classification and taxonomy, Bacterial host response

## Abstract

The laboratory rat emerges as a useful tool for studying the interaction between the host and its microbiome. To advance principles relevant to the human microbiome, we systematically investigated and defined the multitissue microbial biogeography of healthy Fischer 344 rats across their lifespan. Microbial community profiling data were extracted and integrated with host transcriptomic data from the Sequencing Quality Control consortium. Unsupervised machine learning, correlation, taxonomic diversity and abundance analyses were performed to determine and characterize the rat microbial biogeography and identify four intertissue microbial heterogeneity patterns (P1–P4). We found that the 11 body habitats harbored a greater diversity of microbes than previously suspected. Lactic acid bacteria (LAB) abundance progressively declined in lungs from breastfed newborn to adolescence/adult, and was below detectable levels in elderly rats. Bioinformatics analyses indicate that the abundance of LAB may be modulated by the lung–immune axis. The presence and levels of LAB in lungs were further evaluated by PCR in two validation datasets. The lung, testes, thymus, kidney, adrenal and muscle niches were found to have age-dependent alterations in microbial abundance. The 357 microbial signatures were positively correlated with host genes in cell proliferation (P1), DNA damage repair (P2) and DNA transcription (P3). Our study established a link between the metabolic properties of LAB with lung microbiota maturation and development. Breastfeeding and environmental exposure influence microbiome composition and host health and longevity. The inferred rat microbial biogeography and pattern-specific microbial signatures could be useful for microbiome therapeutic approaches to human health and life quality enhancement.

## Abstract (web summary)

The authors defined rat microbial biogeography by re-analyzing publicly available RNA sequencing data from 11 organs of juvenile, adolescent, adult and aged Fisher 344 rats.

## Main

The laboratory rat has been widely used and well examined as a model in a variety of biomedical fields, from cardiovascular diseases to cancer^1^. Recent evidence from the Human Microbiome Project^2^ and The Cancer Genome Atlas pan-cancer microbiome projects^3,4^ suggests that different body sites and disease status feature distinct microbial communities, which have essential roles in human physiology, health and disease. In this Article, we examine the spatial and longitudinal structures of the microbial community in various body compartments and across different life-history stages, to characterize the microbiota landscape of Fischer 344 (F344) rats, with a view to help advance human microbiome research.

The F344 is an inbred laboratory strain of rats that is frequently used in aging, cancer and toxicity studies^5^. Throughout the natural lifespan of F344 rats, 2–104 weeks would be equivalent to 1–3 months to 70–80 years in humans^6^. In F344 male and female rats, weaning normally occurs at week 3 of age, with sexual maturity by week 7 of age. Thus, our microbial discovery cohort, which was based on the Sequencing Quality Control (SEQC) data^7^, consecutively constitutes four major life-history stages of rats: newborns (2 weeks old), adolescents (6 weeks old), adults (21 weeks old) and seniors (104 weeks old). The Biology of Aging Program from the National Institutes of Health has used multiple mammalian and nonmammalian model systems, including F344 rats, to investigate genetics and other aging-related degenerative changes; however, the contribution of microbes in these processes in F344 rats is still unknown.

In mammals, microbial colonization starts in utero and extends throughout the lifespan, particularly in newborn infants who experience rapid microbial community changes. Human placenta harbors a unique low-abundance microbiome composed of commensal bacteria such as *Escherichia coli*, *Prevotella tannerae* and *Neisseria* spp^8^. Maternal–fetal transmission of microbes has taken place long before birth^9^. Newborns are then exposed to microbes from birth, during breastfeeding and through interactions with their surrounding environments that colonize the newborn’s skin^10^, oral cavity^11^, gut^12^, respiratory tract^13^ and other mucosal surfaces^14^. For example, the neonatal skin microbiome is dynamic, site specific and varies from individual to individual^10^. Neonatal oral microbiota is dominated by *Streptococcus* within the Firmicutes phylum^11^. *Bifidobacterium* and *Lactobacillus* spp. are among the first colonizers of the gastrointestinal (GI) tract, representing the pioneer microbial communities in the newborn’s gut^12^. The replacement of breast milk or infant formula with solid foods greatly changes the infant’s microbial composition to resemble an adult-like gut microbiota^15^. Each microbial niche in the body tends to develop and mature both independently and cooperatively, beginning in the first stages of life^13^. Human microbiomes, particularly gut microbiomes, remain stable once established during adulthood^16^. Later in life, elderly individuals are characterized by decreased microbial diversity and shifts in community structure^17^. For laboratory neonatal rodent models, which are more exposed to fecal and environmental contaminants than humans^18^, limited studies have shown that Enterobacteriaceae and *Lactobacillus* are among the most dominant bacteria in newborn mice and rats gut microbiota^19,20^. Similar to studies carried out in humans, adult rodents are typically selected because their body microbial community is more stable.

Translocation of indigenous microbes, such as Enterobacteriaceae, *Lactobacillus* and *Staphylococcus*^19^, from the GI tract to other distant organs via systemic circulation is common in humans and rats^19,21^. Furthermore, microbial bidirectional interactions across multiple organs, including (but not limited to) the gut–brain, lung–brain, gut–liver and gut–lung axes, are important for shaping immune responses and cross-niche microbial interactions^22–26^. The axes can be disrupted by dietary components and the host’s health conditions. For example, in a rat maternal separation model, Donoso et al.^22^ found that maternal separation-induced behavioral despair substantially correlated with gut microbiota changes. A dietary intervention with polyphenols can not only alter gut microbiota composition, but also reverse depressive-like behaviors in the rats. Similarly, olanzapine-induced metabolic dysfunction is associated with the gut–liver axis in rats^24^. Diet-derived intestinal short-chain fatty acids are thought to impact the course of respiratory disorders^25^.

Although the gut microbiome is the largest reservoir of microorganisms in mammals, the current study expands on other organ systems to provide more in-depth analyses on currently unknown microbial biogeography in different niches of the rat body. More specifically, we focused our attention on RNA sequencing (RNA-seq)-derived rat microbial data from 11 organs including brain, lung, heart, liver, muscle, spleen, thymus, kidney, adrenal gland, uterus (females) and testes (males)^7^. We take advantage of this longitudinal data to investigate the tissue-level microbial diversity and community changes during the full lifespan of rats, and aim to identify age-dependent species for future experimental validations. Furthermore, intertissue microbial heterogeneity and microbe–host gene interactions were well examined and assessed. Our aims were to (1) identify core and uncommon microbial species in healthy rats, (2) determine and compare microbial profiles of different anatomical sites over time, (3) define age-dependent microbial species, (4) investigate intertissue microbial heterogeneity and (5) build microbe–host gene interaction networks in F344 rats. In our study, in addition to characterizing the distribution of microbes and their phylogenetic diversities spatially and longitudinally, the inferred lung–immune axis and identification of tissue-specific core species in F344 and Sprague Dawley (SD) rats are useful for establishing future links between microbiome and human health.

## Results

### Core microbial species in two rat strains

**Fig. 1:**
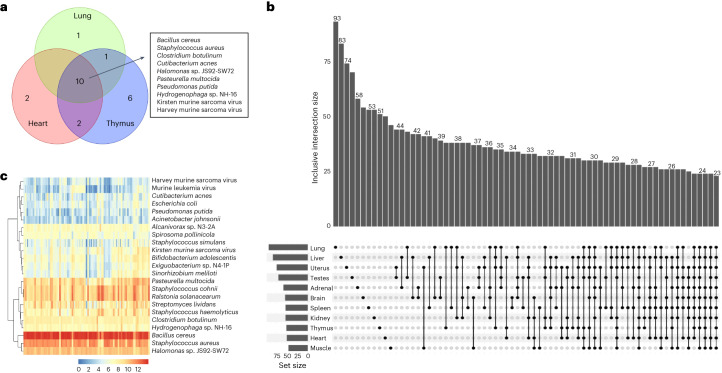
Core microbial species in two rat strains. **a**, Overlapped core species in the lung, heart and thymus of SD and F344 rat strains. The number of overlapped species between the tissues are shown by a Venn diagram. The names of ten common core species between the three organs are shown next to the Venn diagram. **b**, An UpSet plot displays the intersected core species among the 11 tissues in the F344 rats (*n* = 162; adolescent age). Each bar in the bar chart shows a different combination of tissues and the size of inclusive intersected species (values in the *y* axis). The graphical table below the bar chart indicates the corresponding memberships. Each row in the table is 1 of the 11 tissues. The filled black dots and lines show the combination of tissues that have sets intersections. A smaller bar chart on the left side of the graphical table displays the size of elements per set. **c**, A heat map of the abundance of the 23 core species in the F344 rats (*n* = 162; adolescent age). The columns indicate the samples and rows indicate the species. Red color indicates higher abundance and blue represents lower abundance. The samples and species were hierarchically clustered (complete linkage, Euclidean distance).

Core microbial species can be defined as a group of species that are present in all investigated samples, and which might be biologically associated with particular habitats^27^. Lung, heart and thymus were among the three major organs of our initial interest. In the SEQC F344 rat strain (sample size: *n* = 16–17 for lung/heart/thymus; adolescent age; Table 1), and in the SD rat strain (validation data set 1; adolescent age; *n* = 17; Table 2), we identified 12, 14 and 19 overlapped core species in the lung, heart and thymus, respectively (Fig.1a and Supplementary Data 1). *Pelodictyon phaeoclathratiforme*, an environmental species, is the lung-specific core microbial species. Two opportunistic pathogens, namely *Staphylococcus epidermidis* and *Pseudomonas tolaasii* are heart-specific core species. Four Proteobacteria and one Firmicutes, which are widespread in a variety of natural environments, plus one blood virus (avian myeloblastosis virus) are thymus-species cores (Supplementary Data 1). A total of ten common core species were subsequently identified from the three rat organs, which included eight bacterial species in three major phyla (including Firmicutes, Proteobacteria and Actinobacteria) and two murine sarcoma viruses (Fig. 1a and Supplementary Data 1). *Cutibacterium acnes*, *Pasteurella multocida* and *Staphylococcus aureus* are part of the commensal flora of human and animal microbiomes^28^. *Clostridium botulinum* is a foodborne pathogen that causes illness in humans and animals. The Harvey and Kirsten murine sarcoma virus (Ha-MuSV and Ki-MuSV) are two endogenous leukemia viral sequences detected in both tumor and normal tissues in rats^29^. Four of the ten common core species are environmental species, including two soil bacterium (*Bacillus cereus and C.* *botulinum*) and two water sediment-associated microorganisms (*Halomonas* sp. JS92-SW72 and *Hydrogenophaga* sp. NH-16).

**Table 1:** Sample sizes in the discovery dataset^7^ (RNA-seq of F344 rats)

	Developmental stage			
Tissue	Newborn (2 weeks)	Adolescent (6 weeks)	Adult (21 weeks)	Senior (104 weeks)
Adrenal	17	16	16	17
Brain	17	16	16	17
Heart	17	16	17	16
Kidney	18	16	16	16
Liver	16	16	16	18
Lung	18	16	16	16
Muscle	17	17	16	16
Spleen	16	16	17	17
Thymus	16	17	16	17
Testes	8	8	8	8
Uterus	8	8	9	9
Total	168	162	163	167

**Table 2:** Individual characteristics and sample sizes in validation datasets

Species	Individual ID	Age	Gender	BMI (kg/m^2^)	Tissue	Data type	Sample size
**Validation dataset 1**							
SD rat	C1123	7.5 weeks	M	–	Lung, heart and thymus	RNA-seq (lung, heart and thymus) and cDNA (lung)	3
	C1450	7.5 weeks	M	–	Lung, heart and thymus		3
	C1456	7.5 weeks	M	–	Lung, heart and thymus		3
	C1151	7.5 weeks	F	–	Lung and heart		2
	C1451	7.5 weeks	F	–	Lung, heart and thymus		3
	C1460	7.5 weeks	F	–	Lung, heart and thymus		3
**Validation dataset 2**							
Human	A11	41 years	M	51.76	Lung	DNA	1
	A12	33 years	M	30.56	Lung	DNA	1
	A13	41 years	M	32.43	Lung	DNA	1

In the SEQC F344 strain (*n* = 162; adolescent age), we further identified core microbial species for the remaining organs (Supplementary Data 1). An UpSet plot (Fig. 1b) was generated to display the intersections among different core species in different tissue combinations, and the numbers of intersected species are shown in the bar chart. The lung and the muscle have the largest and smallest number of core species, respectively (Fig. 1b). There were 23 common core species, and the 10 species we identified above were all present as cores in different organ systems, suggesting they are important microbial species in rats. Hierarchical clustering of the 23 core species in F344 rats identified two major groups: one with low and one with high abundance (Fig. 1c). Harvey murine sarcoma virus, *C.* *acnes* and *E.* *coli* were among the lowest abundances across all samples, compared with the other core species. The most abundant core species across all rat tissues was *B.* *cereus* (Fig. 1c). Previous studies have shown beneficial effects of *B.* *cereus* on the health of animals^30^, and one *B.* *cereus* var. Toyoi has been authorized to be used as feed supplements in piglet farming^31^. It is possible that *B.* *cereus* in rats were gained from animal feed. The pathogenic potential of *B.* *cereus* has been recognized in humans recently^32^, reinforcing the importance of safe handling practices when working with (laboratory) animals.

### Microbial tissue-level associations and microbial composition and diversity of the four developmental stages

**Fig. 2:**
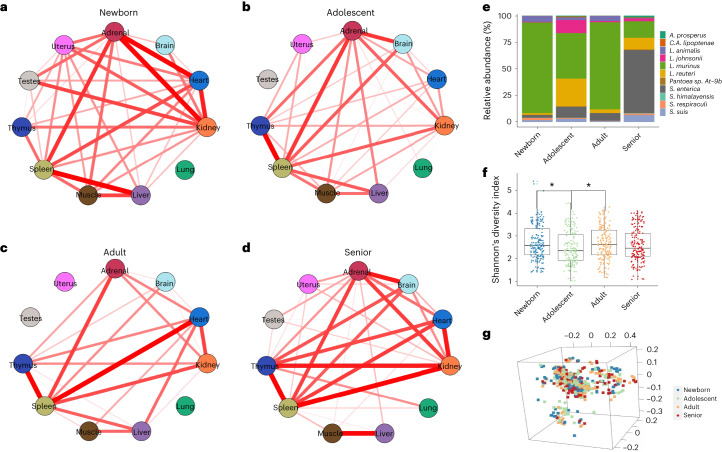
Tissue-level microbial associations and microbial composition and diversity at the four developmental stages. **a**–**d**, Circular correlation networks display the Spearman’s correlations for all pairs of tissue microbiome associations at the newborn (**a**; *n* = 168), adolescent (**b**; *n* = 162), adult (**c**; *n* = 163) and senior (**d**; *n* = 167) stage. The strength of the correlations are proportional to their absolute correlation coefficients, and the color indicates the direction of the correlations (red for positive and blue for negative correlations). Only strong correlations with absolute correlation coefficients greater than 0.5 are shown. **e**, Species-level relative abundance (%; *y* axis) of the 11 stage-related bacteria for each four developmental stages**. f**, Species-level Shannon’s diversity index (*y* axis) at the four stages. Pairwise Wilcoxon rank sum tests with BH correction were used to test for diversity differences between stages. Only statistically significant comparisons (adjusted *P* < 0.05) are marked with a single star (*), and adjusted *P* values <0.01 are marked with two stars (**). **g**, Species-level three-dimensional view PCoA of Bray–Curtis dissimilarity between samples colored in four different colors according to each developmental stages.

We identified a total of 2,829 microbial species in the F344 rat dataset (*n* = 660), spanning 45 unique and unclassified phyla at a 10% prevalence threshold (Supplementary Data 2). Firmicutes and Proteobacteria were the two dominant phyla, altogether ranging from 86.8% in seniors to 89.4% in adolescent rats (Supplementary Fig. 1a and Supplementary Data 3). The other phyla included Actinobacteria, Euryarchaeota and Bacteroidetes (Supplementary Fig. 1a and Supplementary Data 3). Bacillales from the phylum Firmicutes was the most abundant order, followed by Burkholderiales, Pasteurellales and Oceanospirillales from Proteobacteria (Supplementary Fig. 1b and Supplementary Data 3). The average abundances of the microbial species in each tissue type were calculated and used in Spearman correlation analysis to examine associations among tissue microbiomes; only strong correlations with absolute correlation coefficients greater than 0.5 were shown (Fig. 2a–d). Spearman’s correlation coefficients for all tissue combinations were positive, and generally intensive interactions between tissues could be seen in all four developmental stages (Fig. 2a–d). Minor and weak associations were observed in tissues like the lung, uterus and testes at specific stages (Fig. 2a–c), suggesting that they have unique and independent microbiomes compared with other tissues.

Although microbial community compositions at both phylum and order levels showed no significant difference among the four developmental stages (Kruskal’s test; false discovery rate (FDR)-adjusted *P* > 0.05), 11 bacteria were identified to be associated with the stage at the species level as determined by Kruskal’s test adjusted for multiple comparisons (FDR-adjusted *P* < 0.05, Table 3). Seven of the 11 species derived from the Lactobacillales order, three belonged to Enterobacterales and the remaining one species from the order Chromatiales. The Lactobacillales order was further divided into four beneficial *Lactobacillus* and three animal-related *Streptococcus* spp. (Table 3). The relative abundance of *Lactobacillus murinus* was much higher than any other ten age-related species in the newborn, adolescent and adult stages, but significantly decreased in the seniors compared with the newborn and adult rats (pairwise Wilcoxon test adjusted *P* < 0.05; Fig. 2e and Supplementary Data 3). Previous animal studies have shown the protective effects of commensal bacteria *L.* *murinus* against GI diseases^33,34^ and viral respiratory infections^35^. We connected a possible role of *L.* *murinus* in rat normal development and aging processes through bioinformatics analyses, although further experimental validations are needed. *Streptococcus suis* is an important pathogen of pigs, causing severe systemic disease by producing virulence factors that bind host cell molecules^36^. Transmission of *S.* *suis* from feces-contaminated food and water is common among humans and animals^37^. Among the selected 11 species, the relative abundance of *Salmonella enterica*, which is a high-prevalence species of the Enterobacterales order, was significantly increased in the elderly compared with the adolescent rats (pairwise Wilcoxon test adjusted *P* < 0.05; Fig. 2e and Supplementary Data 3). Other age-dependent species that were highly enriched in elderly rats include *Streptococcus himalayensis*, and two environmental species (*Acidihalobacter prosperus* and *Pantoea* sp. At-9b) (Supplementary Data 3). Additionally, the abundance of *Lactobacillus reuteri, Lactobacillus johnsonii* and *Candidatus Arsenophonus lipoptenae* were much higher in adolescent rats than in other stages (Supplementary Data 3). Adolescent rats also showed the significantly lowest microbial alpha diversity at the order and species levels compared with the newborn and adult rats (pairwise Wilcoxon test adjusted *P* < 0.05; Fig. 2f and Supplementary Fig. 1d), although with no significant beta diversity differences compared with the other three developmental stages (Fig. 2g and Supplementary Fig. 1e,f).

**Table 3:** Taxonomy information of the 11 age-dependent species

Adjusted *P* value	Kingdom	Phylum	Class	Order	Family	Genus	Species
0.043	Bacteria	Proteobacteria	Gammaproteobacteria	Chromatiales	Ectothiorhodospiraceae	*Acidihalobacter*	*A.* *prosperus*
0.022	Bacteria	Proteobacteria	Gammaproteobacteria	Enterobacterales	Enterobacteriaceae	*Salmonella*	*S.* *enterica*
0.030	Bacteria	Proteobacteria	Gammaproteobacteria	Enterobacterales	Erwiniaceae	*Pantoea*	*P*. sp. At-9b
0.036	Bacteria	Proteobacteria	Gammaproteobacteria	Enterobacterales	Morganellaceae	*Arsenophonus*	*C.* *A.* *lipoptenae*
0.001	Bacteria	Firmicutes	Bacilli	Lactobacillales	Lactobacillaceae	*Lactobacillus*	*L.* *animalis*
0.014	Bacteria	Firmicutes	Bacilli	Lactobacillales	Streptococcaceae	*Streptococcus*	*S.* *himalayensis*
0.014	Bacteria	Firmicutes	Bacilli	Lactobacillales	Lactobacillaceae	*Lactobacillus*	*L.* *johnsonii*
0.026	Bacteria	Firmicutes	Bacilli	Lactobacillales	Streptococcaceae	*Streptococcus*	*S.* *respiraculi*
0.029	Bacteria	Firmicutes	Bacilli	Lactobacillales	Lactobacillaceae	*Lactobacillus*	*L.* *murinus*
0.037	Bacteria	Firmicutes	Bacilli	Lactobacillales	Streptococcaceae	*Streptococcus*	*S.* *suis*
0.043	Bacteria	Firmicutes	Bacilli	Lactobacillales	Lactobacillaceae	*Lactobacillus*	*L.* *reuteri*

### Lung microbial composition changes and lung–immune axis during the four developmental stages

**Fig. 3:**
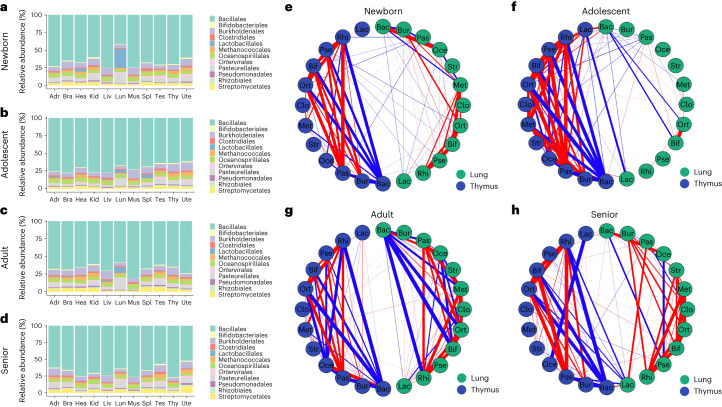
Lung microbial composition changes and lung–immune axis during the four developmental stages. **a**–**d**, Taxa composition bar plots of 11 tissues (adrenal gland (Adr), brain (Bra), heart (Hea), kidney (Kid), liver (Liv), lung (Lun), muscle (Mus), spleen (Spl), testes (Tes), thymus (Thy) and uterus (Ute)) illustrate the microbial relative abundance (%; *y* axis) of the top 12 most abundant orders at the newborn (**a**; *n* = 168), adolescent (**b**; *n* = 162), adult (**c**; *n* = 163) and senior (**d**; *n* = 167) stage. **e**–**h**, Circular correlation networks display the Spearman’s correlations for the top 12 most abundant orders (Bacillales (Bac), Bifidobacteriales (Bif), Burkholderiales (Bur), Oceanospirillales (Oce), *Ortervirales* (Ort), Pasteurellales (Pas), Pseudomonadales (Pse), Rhizobiales (Rhi), Clostridiales (Clo), Lactobacillales (Lac), Methanococcales (Met) and Streptomycetales (Str)) associations between lungs (green) and thymus (dark blue) at the newborn (**e**; *n* = 168), adolescent (**f**; *n* = 162), adult (**g**; *n* = 163) and senior (**h**; *n* = 167) stage. The strength of the correlations are proportional to their absolute correlation coefficients, and the color indicates the direction of the correlations (red for positive and blue for negative correlations). Only strong correlations with absolute correlation coefficients greater than 0.5 are shown.

The above 11 stage-related species were identified at the whole-tissue level, which may not capture the complexity of the tissue-specific microbial changes during development. Therefore, we processed the operational taxonomic unit table at each developmental stage individually to compare the intertissue microbial composition changes (and to identify and compare the age-dependent species for each tissue type, as described later). Bacillales was prominent with a relative abundance of over 40% in order among all tissue groups (Fig. 3a–d and Supplementary Data 4). Liver and muscle generally had the lowest microbial alpha diversity as compared with the other organs across the four developmental stages (pairwise Wilcoxon test, adjusted *P* < 0.05; Supplementary Fig. 2b–e). The pairwise beta diversity comparisons among tissues were all not significant at the four stages (pairwise permutational multivariate analysis of variance (PERMANOVA) adjusted *P* < 0.05; Supplementary Fig. 2f–i).

In newborn rat samples (*n* = 168), pairwise comparisons showed significantly higher Lactobacillales abundance in lungs compared with the other ten tissues (adjusted *P* < 0.05; Fig. 3a and Supplementary Data 4 and 5). The heart showed the second highest relative abundance of Lactobacillales, although not statistically significant compared with the remaining organs (Fig. 3a and Supplementary Data 4 and 5). Lung samples also showed the highest abundance of Pasteurellales in newborn rats, and the pairwise comparisons were all significant except for that with kidney (adjusted *P* < 0.05; Fig. 3a and Supplementary Data 4 and 5).

A significant decrease in Lactobacillales was observed in lungs between newborn and adolescent rat samples (adjusted *P* < 0.05; Fig. 3a,b and Supplementary Fig. 2a). The spleen showed a slightly higher relative abundance of Lactobacillales compared with the lung at the adolescent stage (Fig. 3b and Supplementary Data 4). Other order-level enrichments in adolescent rats include the muscle and uterus samples, which had the first and second highest abundance of Burkholderiales, respectively (Fig. 3b and Supplementary Data 4). Except for the testes and uterus samples, the lung showed a significantly higher abundance of Pasteurellales than other tissues (pairwise Wilcoxon test adjusted *P* < 0.05; Fig. 3b and Supplementary Data 4 and 5).

In adult (*n* = 163) and senior rats (*n* = 167), the most predominant bacteria order in lung tissues compared with any other tissues was Pasteurellales (Fig. 3b–d and Supplementary Data 4). A slight increase in the relative abundance of Lactobacillales was detected in adult lungs compared with adolescent lungs (adjusted *P* = 0.207; Supplementary Fig. 2a). Similar to newborn lungs, adult lungs were significantly enriched with Lactobacillales (pairwise Wilcoxon test adjusted *P* < 0.05; Fig. 3b–c and Supplementary Data 5), with higher Lactobacillales abundance compared with the other the tissues (Supplementary Fig. 2a). In addition, the relative abundances of Streptomycetales in the liver, muscle, lung and uterus of adult rats were much lower than in other tissues (Fig. 3c and Supplementary Data 4).

In elderly samples, the difference in relative abundance of Lactobacillales between the lung and other tissues was no longer significant (pairwise Wilcoxon test adjusted *P* > 0.05); actually the presence of Lactobacillales was almost undetectable across all tissue types (0.21–0.67%) (Fig. 3d and Supplementary Data 4 and 5). Moreover, the lung microbiomes showed distinct separation from other tissues and shifted remarkably in elderly rats compared with younger animals (Supplementary Fig. 2f–i).

The thymus is an important part of the immune system, and thymic involution has central roles in immunosenescence^38^. Multiple microbial taxa were correlated between thymus and lungs during the four developmental stages (Fig. 3e–h). Intratissue microbiome associations were more notable than intertissue associations (Fig. 3e–h), suggesting that the two organ systems have relatively independent microbiomes. Thymus microbiome associations in all four stages were intensive and widespread, and peak lung microbiome associations were reached at the adult stage (Fig. 3e–h). Negative associations of *Lactobacillus* and other taxa between the two organs were observed in the adolescent and senior rats (Fig. 3f,h). To investigate the potential sources of *Lactobacillus* in the lungs, *Lactobacillus* abundance in each tissue type were used in Spearman correlation analysis to examine associations among tissues at the four developmental stages (Supplementary Fig. 3a–d). Lung and liver *Lactobacillus* abundance were strongly and positively associated in the newborn rats (Supplementary Fig. 3a), suggesting they may have a common source of *Lactobacillus*, possibly from the gut. Strong negative *Lactobacillus* associations were found between lungs and thymus in the adolescent rats (Supplementary Fig. 3b). *Lactobacillus* abundance in the spleen, another immune organ, was also negatively associated with the abundance of *Lactobacillus* in the adolescent lung (Supplementary Fig. 3b). Adult lung *Lactobacillus* abundance was independent from other tissues (Supplementary Fig. 3c), and senior lung *Lactobacillus* abundance was negatively associated with *Lactobacillus* abundance in the heart (Supplementary Fig. 3d).

A recent study^23^ showed that a lung lipopolysaccharide-producing microbiome such as Bacillales, Lactobacillales, Bifidobacteriales, Oceanospirillales and Pseudomonadales could regulate brain autoimmunity through the lung–brain axis in adolescents Lewis rats. In the healthy F344 adolescent rat samples used in this study, positive associations of Oceanospirillales, as well as negative associations of Pseudomonadales and Bifidobacteriales, were observed between lungs and brains, indicating the existence of a lung–brain axis in lipopolysaccharide-producing microbial taxa in the healthy state (Supplementary Fig. 3f). The lung–brain axis also exists in other taxa and in other developmental stages (Supplementary Fig. 3e–h). Intratissue microbiome associations were more striking than intertissue interactions, notably in the newborn, adolescent and senior F344 rats, where intensive positive and negative microbiome associations were found in the brains only; in the adult stage, intensive associations were uniquely found in the lungs (Supplementary Fig. 3e–h).

### *Lactobacillus* and *Bifidobacterium* were detected by PCR in healthy lungs

To verify that *Lactobacillus* are present in healthy lungs, both conventional and real-time PCR were performed in adolescent rat and adult human lung tissues. The presence of *Bifidobacterium* was also tested because the two bacteria genera exhibit similar beneficial effects^39^. Genus-specific PCR primers, which target a total of 15 and 30 *Lactobacillus* and *Bifidobacterium* spp., respectively, were taken from ref. ^40^ and used for the detection and quantification of the corresponding bacteria in our study.

Unlike the changes observed in Lactobacillales abundance in rat lungs across different developmental stages (Supplementary Fig. 4a), no changes in abundance were found for Bifidobacteriales in the lungs across the four stages in the discovery dataset (Supplementary Fig. 4b). The average abundance of Lactobacillales in lungs was slightly higher than that of Bifidobacteriales in both the discovery and the validation dataset 1, which was composed of SD rats (although not reaching statistical significance-adjusted *P* > 0.05; Supplementary Fig. 4c,d). The results were based on RNA-seq-derived microbial results from adolescent rats in both datasets. Similarly, the presence of *Lactobacillus* and *Bifidobacterium* was detected by RT–PCR in all six rat lung samples from the validation dataset 1, with no statistical abundance differences between the two bacteria genera (Table 4 and Supplementary Fig. 4e). Furthermore, up to 15 and 30 *Lactobacillus and Bifidobacterium* spp., respectively, were detected in healthy human lungs (validation dataset 2), and *Lactobacillus* bands were generally brighter and more unique than that of *Bifidobacterium* (Supplementary Fig. 4f). Human individual A11 with the highest body mass index (BMI) tested negative for *Lactobacillus*, and had a lung microbiome enriched with different *Bifidobacterium* spp. compared with the other two individuals (Supplementary Fig. 4f).

**Table 4:** Summary of the quantifying microbial abundances by RT–PCR

	Sample	*Repli1 Ct	*Repli2 Ct	*Repli3 Ct	Mean Ct	∆Ct	∆∆Ct	2^ − (∆∆Ct)
***Actb*** **(housekeeping)**	C1123	13.569	13.339	13.873	13.594	–	–	–
	C1450	12.959	12.977	12.951	12.962	–	–	–
	C1456	14.024	13.694	13.873	13.864	–	–	–
	C1151	11.741	14.112	13.704	13.186	–	–	–
	C1451	13.009	13.022	13.046	13.026	–	–	–
	C1460	13.802	13.803	13.887	13.831	–	–	–
***Lactobacillus*** **spp**.	C1123	33.859	32.921	31.866	32.882	19.288	0.440	0.737
	C1450	32.307	30.922	31.172	31.467	18.505	−0.343	1.268
	C1456	29.119	29.097	29.805	29.340	15.476	−3.372	10.351
	C1151	32.109	31.662	34.142	32.637	19.451	0.603	0.658
	C1451	30.818	31.421	32.480	31.573	18.547	−0.301	1.232
	C1460	37.193	34.168	33.787	35.049	21.218	2.370	0.193
***Bifidobacterium*** **spp. (reference)**	C1123	32.284	32.872	32.728	32.628	19.034	0.186	0.879
	C1450	32.381	31.848	32.597	32.275	19.313	0.465	0.724
	C1456	31.133	31.566	32.459	31.719	17.855	−0.993	1.990
	C1151	32.652	32.867	32.980	32.833	19.647	0.799	0.574
	C1451	31.429	32.678	31.910	32.006	18.980	0.132	0.912
	C1460	31.568	32.330	32.366	32.088	18.257	−0.591	1.506
	Reference average	–	–	–	–	18.847	–	–

*Repli, replicate.

### Comparison of age-dependent microbial species among different tissues

Kruskal’s tests adjusted for multiple comparisons at the species level were then carried out to identify the age-dependent microbes for each tissue type. Only the lung, testes, thymus, kidney, adrenal and muscle were found to have 1–52 age-dependent species (adjusted *P* < 0.05; Supplementary Data 6).

**Fig. 4:**
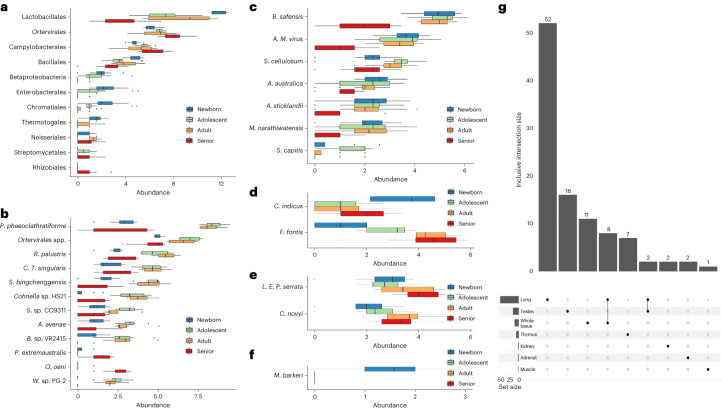
Comparison of age-dependent microbial species among six different tissues. **a**–**f**, Microbial abundance (*x* axis) of selected microbes at the four developmental stages in the lung (**a**; *n* = 18 newborn + 16 adolescent + 16 adult + 16 senior), testes (**b**; *n* = 8 newborn + 8 adolescent + 8 adult + 8 senior), thymus (**c**; *n* = 16 newborn + 17 adolescent + 16 adult + 17 senior), kidney (**d**; *n* = 18 newborn + 16 adolescent + 16 adult + 16 senior), adrenal (**e**; *n* = 17 newborn + 16 adolescent + 16 adult + 17 senior) and muscle (**f**; *n* = 17 newborn + 17 adolescent + 16 adult + 16 senior) samples. **g**, An UpSet plot showing the intersected stage-related microbial species in the six tissues and whole-tissue levels. Each bar in the bar chart shows a different combination of tissues and the size of inclusive intersected species (values in *y* axis). The graphical table below the bar chart indicates the corresponding memberships. Each row in the table is one of the six tissues or whole tissue. The filled black dots and lines show the combination of tissues that have sets intersections. A smaller bar chart on the left side of the graphical table displays the size of elements per set.

A total of 52 species, of which 32 derived from the order of Lactobacillales, together with seven *Bacillus* spp., four *Ortervirales* spp., two *Campylobacterales* spp. and seven other species from seven different bacteria orders were identified to be the age-dependent species in lungs (adjusted *P* < 0.05; Fig. 4a and Supplementary Data 6). Lactobacillales, *Ortervirales*, Campylobacterales and Bacillales were found to be the top four abundant orders associated with lung development and aging (Fig. 4a). The loss of Lactobacillales in elderly lungs (Fig. 4a) agrees well with our previous taxonomy investigation at the order level (Fig. 3a–d). Only lung samples had eight overlapped species with the stage-related microbes identified at the whole/bulk tissue level, and six of them were from the order of Lactobacillales (Fig. 4g and Supplementary Data 6), further indicating that there are strong associations between Lactobacillales spp. and lung microbiota development and maturation.

Five animal-derived *Ortervirales* and 11 environmental species were identified as the age-dependent microbes in testes (adjusted *P* < 0.05; Fig. 4b and Supplementary Data 6). Among them, only *P.* *extremaustralis* and *O.* *oeni* were significantly enriched in the senior testes samples (adjusted *P* < 0.05; Fig. 4b), and the remaining species were significantly enriched in the adolescent and adult samples (adjusted *P* < 0.05; Fig. 4b). Two murine leukemia viruses, namely murine leukemia virus and murine leukemia-related retroviruses, were commonly identified in the lung and testis tissues (Supplementary Data 6).

In thymus samples, we found a total of seven species that changed their abundance during the whole development process (adjusted *P* < 0.05; Fig. 4c and Supplementary Data 6). Among them, there were five environmental species and one animal virus (*B.* *safensis, A.* *australica, A.* *sticklandii, M.* *narathiwatensis, S.* *cellulosum* and Avian myeloblastosis virus), which decreased in elderly samples (Fig. 4c). In addition, *S.* *capitis*, a commensal skin microbe was significantly enriched in the adolescent samples (adjusted *P* < 0.05; Fig. 4c).

Lastly, there were five species that could be involved in the developmental processes of the kidney, adrenal and muscles. More specifically, there was a notable increase and decrease in two environmental microbes, namely *C.* *indicus* and *F.* *fontis*, in newborn kidney samples, respectively (Fig. 4d). Two pathogens, namely *Legionella* endosymbiont of *Polyplax serrata* and *Clostridium novyi*, were notably enriched in the older (adult and elderly) versus young (juvenile and adolescent) adrenal samples (Fig. 4e). *Methanosarcina barkeri*, a methanogenic archaea, was found to be notably decreased in the adolescent, adult and senior stages compared with newborn muscle samples (Fig. 4f).

### Identification of four rat intertissue microbial heterogeneity patterns

**Fig. 5:**
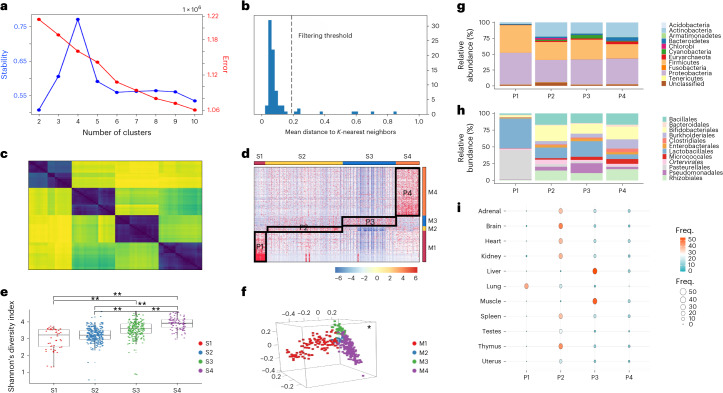
Identification of four rat intertissue microbial heterogeneity patterns. **a**, The *K* selection plot by using the trade-off between solution stability (primary *y* axis; in blue) and solution error (secondary *y* axis; in red). *K* = 4 was selected as the optimal number of clusters as it is highest in stability and has shown relatively lower error rate. **b**, An outlier threshold of ~0.2 was set based on inspecting the histogram of distances between each cluster and its *K*NN. **c**, Clustergram diagnostic heat map of the chosen *K* = 4 showed a high degree of agreement between the replicates with a few outliers. **d**, The microbial abundance heat map with 660 samples in columns (S1–S4) and 357 species in rows (M1–M4), ordered according to their predicted cluster memberships, showed four well-separated microbial heterogeneity patterns (P1–P4). On the heat map, red indicates higher abundance and blue represents lower abundances. **e**, 357 microbial species-level Shannon’s diversity index (*y* axis) of the four patterns. Pairwise Wilcoxon rank sum tests with BH correction were used to test for diversity differences between patterns. Only statistically significant comparisons (*P* < 0.05) are marked with a single star (*), and *P* values <0.01 are marked with two stars (**). **f**, 357 species-level three-dimensional view PCoA of Bray–Curtis dissimilarity between microbial communities colored in four different colors according to each community of microbes. Pairwise multilevel comparisons using adonis with the Bonferroni correction were used to test for beta diversity differences between communities. All pairwise comparisons were significant (*P* < 0.05), and are marked with a single star (*) at the top right position**. g**,**h**, Taxa composition bar plots illustrate the microbial relative abundance (%; *y* axis) of the top 12 most abundant microbes at the phylum (**g**) and the order (**h**) level based on the 357 pattern-specific species’ microbial profile. **i**, A balloon plot to summarize and compare the tissue distributions (rows) for each pattern (columns), where each cell contains a dot whose color and size reflect the magnitude of a numerical value (Freq.).

As no notable beta diversity differences on principal coordinates analysis (PCoA) were observed among tissues over the four courses of aging (pairwise PERMANOVA, adjusted *P* < 0.05; Supplementary Fig. 2f–i), we next performed an unsupervised consensus nonnegative matrix factorization (cNMF)^41^ to group similar tissue microbial communities together based on their shared signature microbes. The 2,829 species-level operational taxonomic unit abundance table of all rat samples (*n* = 660) was used as input for cNMF analysis. cNMF identified a total of four inter-tissue microbial heterogeneity patterns (P1–P4), in which the cluster number *K* = 4 corresponds to the maximum stability in the data (Fig. 5a). The local density filtering threshold was set at ~0.2 based on *K*-nearest neighbor (*K*NN) imputation and the consensus clustergram (Fig. 5b,c). A total of 357 microbial species were subsequently identified as the signatures (or meta-microbe) for each of the four patterns (Supplementary Data 7). The microbial abundance heat map with samples and species, ordered according to their predicted pattern, showed four well-separated microbial heterogeneity patterns (Fig. 5d).

The 660 rat samples were classified into four intertissue subtypes (S1–S4; Supplementary Data 7). S4 had the highest microbial alpha diversity, followed by S3, and the lowest in S1 and S2. Although no significant alpha diversity differences were found between S1 and S2, all other pairwise alpha diversity comparisons between subtypes were significant (pairwise Wilcoxon test adjusted *P* < 0.05; Fig. 5e).

The 357 subtype-specific microbial signatures included 71 different orders belonging to 18 different phyla (Supplementary Data 7). Proteobacteria, Firmicutes, Actinobacteria, Viral and Bacteroidetes were the top five major phyla (Fig. 5g). A total of 117, 17, 40 and 183 microbial signatures for each tissue subtype were used to define the four microbial communities (M1–M4, respectively). All pairwise beta diversity comparisons among the communities were significant (pairwise PERMANOVA, adjusted *P* < 0.05; Fig. 5f).

The four intertissue microbial patterns (P1–P4) each consist of the corresponding tissue subtypes (S1–S4) and microbial communities (M1–M4). S2 contains the largest number of rat samples (310), spanning all 11 different tissue types, but with only one liver and one muscle sample (Fig. 5i and Table 5). There were 17 S2-specific microbial species present in M2, with a substantial higher relative abundance of Bifidobacteriales and *Ortervirales* compared with the other microbial communities (Fig. 5h). Liver and muscle samples were almost exclusively grouped into S3 and S4 (Fig. 5i and Table 5). Similar to S2, S3 had a large sample size (212), spanning all 11 tissues. A total of 40 bacteria species were grouped into M3, which were highly enriched in the 212 samples of S3 (Fig. 5d). The relative abundances of Pseudomonadales and Enterobacterales were much higher in P3 than in any other patterns (Fig. 5d). S4 includes 94 samples but with no lung tissue (Fig. 5i). P4 had higher Burkholderiales, Micrococcales and Clostridiales, and lower Lactobacillales relative abundance compared with the other three patterns (Fig. 5h). S1 is the smallest subtype with 44 samples, and 41 of them come from the lung (Table 5 and Fig. 5i). Lactobacillales and Pasteurellales were the two most dominant microbial signatures in M1 (Fig. 5h).

**Table 5:** The distributions and characteristics of the intertissue subtypes and microbial signatures

	Subtype/microbial community	Number of components	Major types	Number of tissue/order types	No. of correlated genes	Correlated host gene pathways
**P1**	**S1**	44	Lung	3	657	ECM organization Angiogenesis Cell proliferation
	**M1**	117	Lactobacillales Pasteurellales	22		
**P2**	**S2**	310	Testes Brain Thymus	11	>8,000	RNA processing Nervous system Cytokine production
	**M2**	17	Environmental species *Ortervirales*	11		
**P3**	**S3**	212	Liver Muscle	11	653	DNA transcription DNA repair Cell cycle
	**M3**	40	Pseudomonadales Clostridia	20		
**P4**	**S4**	94	No lung	10	2	–
	**M4**	183	Archaea Burkholderiales	45		
**Total**	**Sample**	660	–	11	–	–
	**Microbial signature**	357	–	203		

### Pattern-specific microbe–host gene interactions

We inferred a microbe–host gene interaction network for each of the four intertissue microbial patterns identified in the study. Spearman’s correlation analyses were used to predict putative interactions between microbial species abundances and host gene expressions in each pattern.

**Fig. 6:**
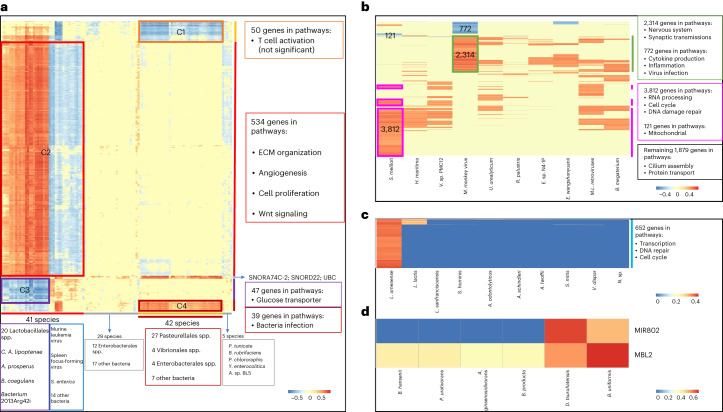
Pattern-specific microbe–host gene interactions. **a**–**d**, Heat maps of the Spearman’s correlation coefficients between the pattern-specific species (in columns) with the host genes (in rows and in human gene symbols) in P1 (**a**), P2 (**b**), P3 (**c**) and P4 (**d**). Red indicates positive correlations and blue represents negative or low-positive correlations. The genes and species were hierarchically clustered (complete linkage, Euclidean distance), and their associated characteristics (such as taxonomic ranks and pathway categories, if any) are shown on the right hand of the corresponding heat map. Gene cluster memberships (C1–C4) are shown in **a** and gene cluster numbers are shown in **b**. *A.* sp. BL5, *Aquimarina* sp. BL5; MBL2, gene mannose-binding lectin 2; MIR802, microRNA 802; *N*. sp., *Novosphingobium* sp. THN1; UBC, gene ubiquitin C.

After filtering, a total of 673 genes were found to be significantly positively/negatively correlated with the abundances of the 117 microbial signatures in P1 (adjusted *P* < 0.05; Supplementary Data 8). The genes and microbes were organized by hierarchical clustering, and their Spearman’s correlation coefficients were visualized in a heat map (Fig. 6a). Absolute correlation coefficients inferior to 0.3, which were considered as weak correlations^42^, were marked as zero to assist better visualization. Although most of the genes seem to be weakly correlated with the majority of microbial signatures, four notable enrichments were depicted in the microbe–host gene heat map (Fig. 6a). Of note, the correlations with genes reflect their combination, rather than the species themselves. More specifically, the largest cluster C2 contained more than 79% (*n* = 534) of the selected 673 genes that were either positively or negatively correlated with 41 microbial species (Fig. 6a and Supplementary Data 8). The 15 *Streptococcus* spp. and five *Lactobacillus* spp. derived from the order of Lactobacillales, and four other species (namely *Candidatus Arsenophonus lipoptenae, Acidihalobacter prosperus, bacterium* 2013Arg42i and *Bacillus coagulans*), as a whole community, were positively correlated with these 534 genes (adjusted *P* < 0.05; Fig. 6a and Supplementary Data 8). Meanwhile, two viruses (murine leukemia virus and spleen focus-forming virus), *S.* *enterica* and the other 14 bacteria were negatively correlated with those host genes. This largest set of genes is significantly involved in extracellular matrix (ECM) organization, angiogenesis, cell proliferation and Wnt signaling pathways (adjusted *P* < 0.05; Supplementary Data 8), which might be coregulated by the microbes and hosts. The remaining three clusters of genes were associated with bacterial infection (C4), glucose transporters (C3) and T cell activation (C1, although not significant-adjusted *P* > 0.05) pathways and processes (Supplementary Data 8).

The 17 microbial species in P2 were significantly associated with a sum of 9,371 host genes (adjusted *P* < 0.05), while ~40% of these microbes each correlated with less than 200 genes. We filtered out microbes with fewer than 200 correlated genes, and the 0.3 absolute correlation coefficient cutoff was applied to remove weakly correlated genes, resulting in a microbe–host gene heat map of ten species and 8,780 host genes (Fig. 6b and Supplementary Data 8). The heat map showed that 3,812 and 2,314 genes were significantly positively correlated with the abundance of *S.* *meliloti* and Mason–Pfizer monkey virus, respectively, while 121 and 772 genes were significantly negatively correlated with these microbial species, respectively (adjusted *P* < 0.05; Fig. 6b and Supplementary Data 8). RNA processing, cell cycle and DNA damage repair-related pathways were significantly overrepresented in the 3,812 genes that were positively correlated with *S.* *meliloti*. The 121 genes negatively correlated with *S.* *meliloti* were enriched in mitochondrial related pathways (adjusted *P* < 0.05; Supplementary Data 8). Mason–Pfizer monkey virus seems to be positively associated with 2,314 host genes in pathways of nervous system and synaptic transmissions. The 772 negatively correlated genes were involved in various cytokine production, inflammation and virus infection-related pathways (adjusted *P* < 0.05; Supplementary Data 8). The remaining positively correlated 1,879 genes, which were partially shared between different microbes including murine leukemia-related retroviruses, were enriched with cilium assembly, cilium movement and protein transport-related pathways (Supplementary Data 8). Cilium-related genes and pathways were also positively correlated with the abundance of *Exiguobacterium* sp. N4-1P.

Gut-derived bacteria *Lachnoanaerobaculum umeaense* was the most significantly positively associated with 652 host genes in P3 (adjusted *P* < 0.05; Fig. 6c and Supplementary Data 8). Kyoto Encyclopedia of Genes and Genomes (KEGG) and Gene Ontology (GO) enrichment analysis indicated that these genes were involved in pathways such as transcription, DNA repair and cell cycle (adjusted *P* < 0.05; Supplementary Data 8). The RNA-binding gene *RBM12B* was positively correlated with another five bacteria species in P3 including *Lactobacillus sanfranciscensis*, *Acinetobacter lwoffii*, *Acinetobacter schindleri*, *Actinomyces odontolyticus* and *Staphylococcus hominis* (adjusted *P* < 0.05; Supplementary Data 8). There were 72 genes that had correlations above 0.3 with the abundance of *Lactococcus lactis*, and which were enriched for cell cycle-related pathways (adjusted *P* < 0.05; Supplementary Data 8). Lastly, only the soil bacteria *Nocardioides* sp. MMS17-SY207-3 was found to be positively correlated with the *TMCC1* gene with unknown function.

Among the 183 microbial signatures in P4, only *Bacteroides uniformis* and pathogen *Delftia tsuruhatensis* each had significant positive correlations with host genes (adjusted *P* < 0.05; Fig. 6d and Supplementary Data 8). More specifically, *MBL2*, which is a component of innate immunity^43^, was found to be positively correlated with the abundance of *B.* *uniformis* and *D.* *tsuruhatensis* in our study. *MIR802*, a glucose metabolism regulator^44^, was more positively correlated with the abundance of *D.* *tsuruhatensis*.

## Discussion

Although rats are commonly used in laboratory settings, little information is available regarding the composition and diversity of their microbial communities. In our study, we systematically investigated the spatial and longitudinal microbial patterns in different body compartments of healthy rats and at different developmental stages. Across the four major life-history stages of rats, we found lung microbial composition changes in taxa such as Lactobacillales and *Ortervirales* (Supplementary Fig. 5), and that liver and muscle exhibit the lowest microbial alpha diversity as compared with other organ systems. The lung, testes, thymus, kidney, adrenal and muscle microbial habitats were found to have 1–52 species that changed their abundances as the rats got older. Moreover, potential negative correlations between the abundance of Lactobacillales and immune system maturation during lung aging were inferred from our study.

Species in the order of Lactobacillales are commonly called lactic acid bacteria (LAB). LAB are a group of Gram-positive lactose fermenters, which have long been known for contributing to the health-promoting properties of milk. Previous studies have shown that microbial colonization of mucosal intestinal tissues during the suckling period shapes the infant’s immune system^14^. Accumulating evidence indicates that, at this stage, microbes (that is, LAB and *Bifidobacterium*) are transmitted from mother to offspring, mainly through breast milk^14,45,46^. In addition to fundamental nutrients and bioactive compounds, human breast milk features a unique microbiome, including mutualist, commensal and probiotic potential bacteria species^47^. The Food and Agriculture Organization of the United Nations and the World Health Organization defines probiotics as ‘live microorganisms which when administered in adequate amounts confer a health benefit on the host’^48^. Thus, previous studies have established a link between immune-enhancing properties and LAB in the gut. In our current study, we found members of immune-related LAB (that is, *L.* *murinus* and *L.* *animalis*), were positively correlated with a large number of host genes involved in cell migration and proliferation, especially in the lungs. A gradual decline in lung LAB abundance was strongly associated with older age (Supplementary Fig. 5), suggesting that LAB could be potentially used as an anti-aging probiotics.

Following this finding, we sought to identify more microbial signatures that reflect the intertissue microbial heterogeneity, irrespective of the developmental stages, in this spatially and temporally heterogeneous data. Compared with core species, uncommon and rare microbes were less observed, but their overall numbers was still considerable with important ecological roles individually or in groups^49^. We have previously used a microbial prevalence threshold at 1% to eliminate rare and potentially contaminated species in two human-associated microbiome studies^50,51^. Given that rodent microbiomes are more likely to be contaminated with fecal matter, and more than two technical replicates are available in the discovery dataset, we increased the threshold to 10% to mitigate the effects of contamination. We initially used the beta diversity for estimating (dis)similarity between microbial communities, and found minor and low beta diversities, which supports the high similarities in tissue-level microbiomes. The lung microbiome was distinct from other tissue microbiomes, only at specific taxa such as Lactobacillus and Pasteurellales. Unsupervised clustering was then used to automatically group similar communities together from their shared signature microbes. On the basis of the 2,829 species identified as global-level microbiome constituents, four intertissue microbial heterogeneity patterns (P1–P4) were identified in an unsupervised fashion (Supplementary Fig. 5), with a total of 357 pattern-specific microbial signatures among the different patterns. The 357 microbial signatures include 71 different orders in 18 different phyla. Proteobacteria, Firmicutes, Actinobacteria, Viral and Bacteroidetes were the top five most abundant phyla. There were 12 Halobacteria in the 357 microbial signatures, which are members of the Archaea domain and are capable of surviving in both low and high salt environments^52^. Using culture-dependent and culture-independent techniques, various halophilic archaea have been identified in salted food products such as fish sauce and table olives^53^. Moreover, the existence of enteric halophilic archaea in the human microbiome is now generally accepted^54,55^. Thus, it is likely that the *Halobacteria* spp. we identified are food-borne microbial species in rats. The 12 Halobacteria, together with another 2 Archaea species, namely *Methanocaldococcus infernus* and *Candidatus Nitrosocosmicus franklandus*, were all microbial signatures from the P4. Four *Ortervirales* spp. plus one *Leucania separata nucleopolyhedrovirus* from P1 and P2, were the only five viral species in the 357 signatures. Microbial signatures in each pattern were selected to investigate the potential microbe–host gene interactions. Since Archaea species account for smaller proportions, and have fewer correlations with host genes compared with the remaining signatures, we focused our attention on the composition and diversity of 338 bacteria and five viral species in each pattern.

More than 93% of the samples in P1 were lung samples (Supplementary Fig. 5). The relative proportion of Lactobacillales in Firmicutes and Pasteurellales in Proteobacteria in P1 account for more than 85% of all 357 microbial species. The relative abundance of Actinobacteria was notably decreased in P1 compared with the remaining three patterns. P2 and P3 contained all 11 tissue types, and no lung samples were found in P4. P2 was the largest pattern representing 41.1% (*n* = 310) of all rat samples, but only 17 species were considered as markers for this pattern. The relative abundance of Bifidobacteriales and *Ortervirales* in P2 were much higher than in the other patterns. Pseudomonadales and Enterobacterales were notably increased in P3 compared with the other patterns. P4 had higher abundance of Burkholderiales, Micrococcales and Clostridiales compared with the other patterns. Microbial signatures in each pattern were mostly positively correlated with different host genes in different metabolic and biological functions. For example, 20 LAB and four other species in P1 were correlated with genes involved in ECM organization, cell migration and proliferation signaling pathways. *L.* *umeaense* in P3 was positively associated with DNA transcription and cell cycle. *B.* *uniformis* in P4 was associated with innate immune signaling. The microbial signatures in P2 largely came from environmental sources such as soil and water sediment. Most environmental species are free living and widely distributed in multiple habitats, which are capable of colonizing and/or causing infections in mammals^56–58^. Compared with germ-free rats, conventional rats are exposed to enriched environments. Thus, it is not surprising that there are several environmental microorganisms within the 23 core species identified in the study. Of particular interest are *S.* *meliloti* and *Exiguobacterium* sp. N4-1P; they are two of the microbial signatures in P2 that showed high abundance in the P2 host samples but low abundance in most liver and muscle tissues. The majority of the correlated host genes and pathways for the two species in P2 samples were positive. For example, *S.* *meliloti* was notably positively correlated with thousands of genes involved in RNA processing and DNA damage repair. Cilium-related genes and signaling pathways were positively correlated with the abundance of *Exiguobacterium* sp. N4-1P. Therefore, species from environmental sources contribute to host microbial diversity and strongly interact with host genes in various cellular functions.

Our study has limitations, including a lack of controls and paired GI samples. Controls for laboratory and experimental contaminants are critical in microbiome studies involving low-biomass samples. However, we did not include control samples because we did not consider the issue at the time of experiments. In the public F344 database, we used a relatively higher prevalence threshold (10%) to mitigate the effects of contamination. Some rare microbial species may therefore have been present but not detected. The GI microbiome is among the most studied and best characterized microbial niche to date. GI microbiota are distinct between wild and laboratory-kept rodents^59^. In laboratory settings, a wide variety of symbiotic microorganisms are present in the rat digestive tract and the gut microbiome is primarily composed of Firmicutes and Bacteroidetes phyla^60^. Previous studies using 16S amplicon sequencing and RT–PCR have shown that LAB (within the Firmicutes phylum) are the most abundant bacteria in the rat gut microbiota^40,61,62^. In addition, LAB have a protective effect, and are one of the most predominant microbes in the feces of the breast-fed neonatal rats^19^. Laboratory rodent diets can be a major source of microbial communities in the GI tract^63^. Rats are omnivorous, eating a variety of plant and animal food items. Grain-based and purified diets represent two of the major commercially made diets used in laboratory rodent studies^63,64^. Grain-based diets contain cereal grains, wheat middling, animal byproducts, many nonnutrients and contaminants, and show batch-to-batch variability. By contrast, purified diets are made with highly refined ingredients, and have many other advantages that make them a more suitable choice^63^. Various prebiotic and probiotic supplements have been tested for modulating the gut microbiota in healthy rats, often leading to an increase in LAB^65^. It is possible that LAB are transmitted from the gut to the lungs via the gut–lung axis. Our future rat studies should be carefully designed to include GI samples and use purified diets with and without supplements. Other limitations in our study include inadequate evaluation of the SD rat microbiomes, and the use of a retrospective and largely single-center study design.

For ethical reasons, stage-wise multisampling in humans is mostly not feasible. Consequently, rodents may serve as a valuable tool to enhance sampling and handling in microbiome studies^66^. Unlike humans, laboratory rats are small nocturnal caged animals; therefore, gaps and challenges exist in translating research findings gained from rat studies to human situations. Moreover, it is estimated that more than 111 million of mice and rats are killed in US laboratories each year^67^. Understanding laboratory rodents’ social life^68^ and maintaining an adequate amount of ‘good’ bacteria in lungs is vital to overall health and animal welfare^69^.

## Conclusions

We systematically investigated the spatial and longitudinal structures of the microbial community in 11 body compartments and across four life-history stages of healthy F344 rats. The abundance of LAB in the lungs declined from breastfed newborn to adolescence/adult and was below detectable levels in elderly rats. Bioinformatics analyses indicate that the abundance of LAB may be modulated by the lung–immune axis. The lung, testes, thymus, kidney, adrenal and muscle microbial habitats had 1–52 species that changed their abundances as the rats got older. The liver and muscle exhibit the lowest microbial alpha diversity compared with other organ systems across the four major developmental stages. Four intertissue microbial heterogeneity patterns were identified and characterized by integrating microbial abundance with host transcriptomic data. Breastfeeding and environmental exposure influenced microbiome composition and host health and longevity. The inferred rat microbial biogeography, and the 357 pattern-specific microbial signatures, especially the LAB, should be useful for advancing human microbiome research.

## Methods

### Ethics approval and consent to participate

For human data: the study was performed in accordance with the Declaration of Helsinki. The Ethics Committees of Stanford Health Care approved the study protocol. For animal data: the study had ethical approval from the Ethics Committee of the VA Palo Alto institutional animal care and use committee (NIM2008).

### Consent for publication

All three human participants signed a written informed consent.

### Human lung tissue collection, DNA extraction and PCR

Our study included three male participants who were lung donors at the Stanford Hospital in the year 2022, and all participants signed a written informed consent. The study was performed in accordance with the Declaration of Helsinki. The Ethics Committees of Stanford Health Care approved the study protocol. Demographic data including age, ethnicity and BMI are summarized in Table 2. Right lung tissues were sampled and embedded in formalin-fixed paraffin-embedded blocks. Genomic DNA was isolated from archived formalin-fixed paraffin-embedded using the DNeasy Blood and Tissue Kit (Qiagen) following the manufacturer’s protocols. DNA concentration and purity were measured by a Nanodrop ND-1000 spectrophotometer.

The DNA was then subjected to conventional PCR using JumpStart REDTaq ReadyMix PCR Reaction Mix (Sigma) and genus-specific primers for bacteria detection in human lung tissues. The primers were taken from Delroisse et al.^40^, which allow for the detection of a wide range of *Lactobacillus* spp. (15 species) and *Bifidobacterium* spp. (30 species) through PCR amplification. Only forward and reverse primers were used and synthesized by Elim Biopharmaceuticals. PCR reactions without primer served as negative controls. The PCR amplification protocol was described in ref. ^40^, and PCR products were evaluated by 2% agarose gel electrophoresis.

### Rat lung tissue collection, real-time PCR and RNA-seq

Six SD rats, including three males and three females, from Charles River were housed at the VA Palo Alto Health Care System facility under a 12 h light/dark cycle in standard conditions. Rats were fed with a normal grain-based chow diet and water ad libitum. The rats were sacrificed at 7.5 weeks to collect lung, heart and thymus tissues. Tissues were snap frozen in liquid nitrogen immediately after excision and stored at –80 °C. Total RNA was extracted using the RNeasy Plus Mini Kit (Qiagen) following the manufacturer’s instructions. The study had ethical approval from the Ethics Committee of the VA institutional animal care and use committee (NIM2008).

RNA qualities were measured by Agilent 2100 Bioanalyzer. The RNA integrity numbers (RINs) of the 12 RNA samples for lungs and hearts were all above 7.5. RIN scores for the five thymus samples varied from 4.6–7.0 (average RIN of 6.3; one sample with low RIN was excluded). Reverse transcription for lung samples was performed using the High Capacity cDNA Reverse Transcription Kit with RNAse Inhibitor and random primers (Applied Biosystems) under the manufacturer’s recommended conditions. Single-stranded complemntary DNAs were then subjected to SYBR Green RT–PCR using primers from ref. ^40^ for the detection and quantification of *Lactobacillus* and *Bifidobacterium* spp. in rat lungs. The rat *Actb* gene was used as the housekeeping reference, and reactions without cDNA template served as negative controls. RT–PCR reactions (all samples were run in triplicate) were carried out on the QuantStudio™ 7 Flex system (Applied Biosystems), and the results were calculated by the delta–delta Ct (∆∆Ct) method.

PolyA-enriched, 150 bp paired-end mRNA-Seq libraries were prepared and high-throughput sequencing was performed on Illumina NovaSeq platform by Novogene. Sequencing data have been deposited to the European Nucleotide Archive (ENA) under accession number PRJEB57257.

### RNA-seq data acquisition and processing

Raw RNA-seq data were downloaded from the ENA database under accession number PRJNA238328. The longitudinal data was generated by the SEQC consortium from 11 organs of both sexes of F344 rats^7^. More specifically, the 11 organs were brain, lung, heart, liver, muscle, spleen, thymus, kidney, adrenal gland, uterus and testes; four developmental stages from newborns (2 weeks old), adolescents (6 weeks old), adults (21 weeks old) to seniors (104 weeks old) were included in our analyses. Rats were fed with a cereal-based NIH-31 diet (ad libitum). Details on housing, necropsy, organ collection and sequencing can be found in the original article^7^. A total of 660 samples were collected and sequenced from 32 healthy female and male rats. which were pair housed (two per cage)^7^. There were four females and four males in each organ and age group with two to four technical replicates. The sample sizes and grouping categories are summarized in Table 1.

Sequences including the PRJNA238328 and our newly generated RNA-seq data were mapped to the Rnor 6.0 reference genome (Ensembl release 104) using STAR (v2.7.9a). Uniquely mapped reads were counted to each gene and converted to transcripts per million for subsequent correlation analysis. The unmapped reads were subjected to re-mapping against a Kraken database, which was built from complete Bacterial, Viral and Archaeal reference genomes from RefSeq^51^.

Kraken (v2.0.8-beta) species-level taxonomic assignments for all samples were combined, and the phyloseq R package (v1.36.0) (ref. ^70^) was used for the phylogenetic and diversity analyses. Core microbial species were identified using a prevalence threshold of 1.0 (presence in all samples). Rare species that were not present in at least one read count in 10% of the samples were removed from further consideration. Median sequencing depth normalization was used for correcting sequencing depth differences between samples. Microbial count data were log2-transformed before statistical analyses. The scripts in this study are available in: https://github.com/lz711/Rat-Microbiome.

### Statistical analyses of microbial community data

Differences in microbial communities within groups (alpha diversity) and between groups (beta diversity) were evaluated and compared at phylum, order and species levels, respectively. Alpha diversity within a sample was measured using the Shannon index, and the diversity differences between groups were assessed using the pairwise Wilcoxon rank sum tests. Benjamini–Hochberg (BH) correction was used to adjust for multiple comparisons. An adjusted *P* value <0.05 was considered statistically significant. PCoA and permutational multivariate analysis of variance (PERMANOVA) were performed using the Bray–Curtis distance with 999 permutations to evaluate differences in beta diversity of microbial communities. The analysis was carried out using adonis in the vegan package^71^. Diversity differences between groups were assessed by the pairwise multilevel comparison using adonis with the Bonferroni correction, and *P* values of less than 0.05 were considered significant. The PCoA was visualized interactively in three-dimensions with the rgl package^72^.

Kruskal’s test with FDR adjustment for multiple comparisons was used to evaluate the correlation between microbial species and grouping variables, such as age groups and tissue groups. Statistical significance was set as *P* < 0.05.

### Identification of rat intertissue microbial heterogeneity patterns

Microbial species that were present in at least one read count in 10% of the total samples (*n* = 660; discovery dataset) were used for the subsequent unsupervised clustering. cNMF (v1.3), which enables the identification of biologically meaningful patterns in high-dimensional datasets^41^, was applied with Kullback–Leibler divergence to identify the intertissue microbial heterogeneity patterns and signatures in the study.

cNMF clustering was run on values of *K* from 2 to 10, and the value of *K* that maximizes stability was selected as the optimal cluster number. Outliers were filtered out based on *K*NN imputation, and the ‘Max’ method^73^ was used for microbial signature selection for each pattern.

### Construction of microbe–host gene interaction networks

A microbe–host gene interaction network consists of a collection of microbial species and their correlated host genes. Spearman’s correlation analyses were performed to examine the correlations between microbial abundance of each species in each pattern with host transcriptomes. FDR-adjusted *P* values of <0.05 were considered statistically significant. The union of all selected significantly correlated genes in each pattern were used to construct a microbe–host gene heat map, respectively, for each pattern. Hierarchical clustering of microbes and genes was done using the complete method with Euclidean distances (default settings) in the pheatmap package in R^74^. Absolute Spearman’s correlation coefficients no less than 0.3 were considered further for the following functional enrichment analysis.

### Functional enrichment analysis

BiomaRt^75^ was used to perform gene nomenclature and rat–human ortholog conversions. Kyoto Encyclopedia of Genes and Genomes and Gene Ontology enrichment analysis of the selected genes (in human gene symbols) significantly correlated with microbial species were analyzed by using Enrichr^76^, and the resulting adjusted *P* values of smaller than 0.05 were considered significant.

### Reporting summary

Further information on research design is available in the Nature Portfolio Reporting Summary linked to this article.

## Online content

Any methods, additional references, Nature Portfolio reporting summaries, source data, extended data, supplementary information, acknowledgements, peer review information; details of author contributions and competing interests; and statements of data and code availability are available at 10.1038/s41684-023-01322-x.

### Supplementary information

Supplementary Information Supplementary Figs. 1–5.

Reporting Summary

Supplementary Data Supplementary Data 1–8.

## Data Availability

The discovery dataset is available from the ENA database under accession number PRJNA238328. The newly generated rat data have been deposited to the ENA database under accession number PRJEB57257.

## References

[1] Smith, J. R., Bolton, E. R. & Dwinell, M. R. The rat: a model used in biomedical research. *Methods Mol. Biol.*10.1007/978-1-4939-9581-3_1 (2019). 10.1007/978-1-4939-9581-3_1 31228150

[2] Human Microbiome Project Consortium (2012). Structure, function and diversity of the healthy human microbiome. Nature.

[3] Poore GD (2020). Microbiome analyses of blood and tissues suggest cancer diagnostic approach. Nature.

[4] Zhao L, Cho WCS, Luo J-L (2022). Exploring the patient-microbiome interaction patterns for pan-cancer. Comput. Struct. Biotechnol. J..

[5] Holmes DJ (2003). F344 rat. Sci. Aging Knowledge Environ..

[6] Kwekel JC, Desai VG, Moland CL, Branham WS, Fuscoe JC (2010). Age and sex dependent changes in liver gene expression during the life cycle of the rat. BMC Genomics.

[7] Yu, Y. et al. A rat RNA-seq transcriptomic BodyMap across 11 organs and 4 developmental stages. *Nat. Commun.*10.1038/ncomms4230 (2014). 10.1038/ncomms4230 PMC3926002 24510058

[8] Aagaard K (2014). The placenta harbors a unique microbiome. Sci. Transl. Med..

[9] Stinson LF, Boyce MC, Payne MS, Keelan JA (2019). The not-so-sterile womb: evidence that the human fetus is exposed to bacteria prior to birth. Front. Microbiol..

[10] Younge NE, Araújo-Pérez F, Brandon D, Seed PC (2018). Early-life skin microbiota in hospitalized preterm and full-term infants. Microbiome.

[11] Kageyama S (2019). Transition of bacterial diversity and composition in tongue microbiota during the first two years of life. mSphere.

[12] Bäckhed F (2015). Dynamics and stabilization of the human gut microbiome during the first year of life. Cell Host Microbe.

[13] Reyman M (2021). Microbial community networks across body sites are associated with susceptibility to respiratory infections in infants. Commun. Biol..

[14] Gensollen T, Iyer SS, Kasper DL, Blumberg RS (2016). How colonization by microbiota in early life shapes the immune system. Science.

[15] Laursen MF, Bahl MI, Michaelsen KF, Licht TR (2017). First foods and gut microbes. Front. Microbiol..

[16] Faith JJ (2013). The long-term stability of the human gut microbiota. Science.

[17] Salazar N, Valdés-Varela L, González S, Gueimonde M, de Los Reyes-Gavilán CG (2017). Nutrition and the gut microbiome in the elderly. Gut Microbes.

[18] Inoue R, Ushida K (2003). Development of the intestinal microbiota in rats and its possible interactions with the evolution of the luminal IgA in the intestine. FEMS Microbiol. Ecol..

[19] Yajima M (2001). Bacterial translocation in neonatal rats: the relation between intestinal flora, translocated bacteria, and influence of milk. J. Pediatr. Gastroenterol. Nutr..

[20] Mirpuri J (2014). Proteobacteria-specific IgA regulates maturation of the intestinal microbiota. Gut Microbes.

[21] Berg RD (1995). Bacterial translocation from the gastrointestinal tract. Trends Microbiol.

[22] Donoso F (2020). Polyphenols selectively reverse early-life stress-induced behavioural, neurochemical and microbiota changes in the rat. Psychoneuroendocrinology.

[23] Hosang L (2022). The lung microbiome regulates brain autoimmunity. Nature.

[24] Luo C (2021). Coadministration of metformin prevents olanzapine-induced metabolic dysfunction and regulates the gut–liver axis in rats. Psychopharmacology.

[25] Enaud R (2020). The gut–lung axis in health and respiratory diseases: a place for inter-organ and inter-kingdom crosstalks. Front. Cell Infect. Microbiol..

[26] Zhao L, Luo J-L, Ali MK, Spiekerkoetter E, Nicolls MR (2023). The human respiratory microbiome: current understandings and future directions. Am. J. Respir. Cell Mol. Biol..

[27] Hamady M, Knight R (2009). Microbial community profiling for human microbiome projects: tools, techniques, and challenges. Genome Res..

[28] Foster, T. Staphylococcus. In *Medical Microbiology* 4th edn Ch. 12 (ed. Baron, S.) (University of Texas Medical Branch at Galveston, 1996). 21413252

[29] Anderson GR, Robbins KC (1976). Rat sequences of the Kirsten and Harvey murine sarcoma virus genomes: nature, origin, and expression in rat tumor RNA. J. Virol..

[30] Trapecar M (2011). The use of a porcine intestinal cell model system for evaluating the food safety risk of *Bacillus cereus* probiotics and the implications for assessing enterotoxigenicity. APMIS.

[31] Altmeyer S, Kröger S, Vahjen W, Zentek J, Scharek-Tedin L (2014). Impact of a probiotic *Bacillus cereus* strain on the jejunal epithelial barrier and on the NKG2D expressing immune cells during the weaning phase of piglets. Vet. Immunol. Immunopathol..

[32] Messelhäußer, U. & Ehling-Schulz, M. *Bacillus cereus*—a multifaceted opportunistic pathogen. *Curr. Clin. Microbiol. Rep.*10.1007/s40588-018-0095-9 (2018).

[33] Isani M (2018). *Lactobacillus murinus* HF12 colonizes neonatal gut and protects rats from necrotizing enterocolitis. PLoS ONE.

[34] Hu J (2022). *Lactobacillus murinus* alleviate intestinal ischemia/reperfusion injury through promoting the release of interleukin-10 from M2 macrophages via Toll-like receptor 2 signaling. Microbiome.

[35] Yildiz S (2020). Respiratory tissue-associated commensal bacteria offer therapeutic potential against pneumococcal colonization. eLife.

[36] Johansson MM (2020). The binding mechanism of the virulence factor *Streptococcus suis* adhesin P subtype to globotetraosylceramide is associated with systemic disease. J. Biol. Chem..

[37] Wertheim HFL, Nghia HDT, Taylor W, Schultsz C (2009). *Streptococcus suis*: an emerging human pathogen. Clin. Infect. Dis..

[38] Ponnappan S, Ponnappan U (2011). Aging and immune function: molecular mechanisms to interventions. Antioxid. Redox Signal..

[39] Eastwood J, Walton G, Van Hemert S, Williams C, Lamport D (2021). The effect of probiotics on cognitive function across the human lifespan: a systematic review. Neurosci. Biobehav. Rev..

[40] Delroisse J-M (2008). Quantification of *Bifidobacterium* spp. and *Lactobacillus* spp. in rat fecal samples by real-time PCR. Microbiol. Res..

[41] Kotliar, D. et al. Identifying gene expression programs of cell-type identity and cellular activity with single-cell RNA-seq. *eLife*10.7554/elife.43803 (2019). 10.7554/eLife.43803 PMC6639075 31282856

[42] Ratner B (2009). The correlation coefficient: its values range between +1/−1, or do they?. J. Target. Meas. Anal. Mark..

[43] Bernig T (2004). Sequence analysis of the mannose-binding lectin (MBL2) gene reveals a high degree of heterozygosity with evidence of selection. Genes Immun..

[44] Kornfeld J-W (2013). Obesity-induced overexpression of miR-802 impairs glucose metabolism through silencing of Hnf1b. Nature.

[45] Martín R (2003). Human milk is a source of lactic acid bacteria for the infant gut. J. Pediatr..

[46] Fehr K (2020). Breastmilk feeding practices are associated with the co-occurrence of bacteria in mothers’ milk and the infant gut: the CHILD cohort study. Cell Host Microbe.

[47] Lyons KE, Ryan CA, Dempsey EM, Ross RP, Stanton C (2020). Breast milk, a source of beneficial microbes and associated benefits for infant health. Nutrients.

[48] Hill C (2014). Expert consensus document. The International Scientific Association for Probiotics and Prebiotics consensus statement on the scope and appropriate use of the term probiotic. Nat. Rev. Gastroenterol. Hepatol..

[49] Banerjee S, Schlaeppi K, van der Heijden MGA (2018). Keystone taxa as drivers of microbiome structure and functioning. Nat. Rev. Microbiol..

[50] Zhao L, Cho WC, Nicolls MR (2021). Colorectal cancer-associated microbiome patterns and signatures. Front. Genet..

[51] Zhao L (2021). Characterization of the consensus mucosal microbiome of colorectal cancer. NAR Cancer.

[52] Andrei A-Ş, Banciu HL, Oren A (2012). Living with salt: metabolic and phylogenetic diversity of archaea inhabiting saline ecosystems. FEMS Microbiol. Lett..

[53] Lee H-S (2013). Diversity of halophilic archaea in fermented foods and human intestines and their application. J. Microbiol. Biotechnol..

[54] Oxley APA (2010). Halophilic archaea in the human intestinal mucosa. Environ. Microbiol..

[55] Lurie-Weinberger MN, Gophna U (2015). Archaea in and on the human body: health implications and future directions. PLoS Pathog..

[56] Zhou D (2016). Exposure to soil, house dust and decaying plants increases gut microbial diversity and decreases serum immunoglobulin E levels in BALB/c mice. Environ. Microbiol..

[57] Blum WEH, Zechmeister-Boltenstern S, Keiblinger KM (2019). Does soil contribute to the human gut microbiome?. Microorganisms.

[58] Haque M, Sartelli M, McKimm J, Abu Bakar M (2018). Health care-associated infections—an overview. Infect. Drug Resist..

[59] Bowerman KL (2021). Effects of laboratory domestication on the rodent gut microbiome. SME Communications.

[60] Lleal, M. et al. A single faecal microbiota transplantation modulates the microbiome and improves clinical manifestations in a rat model of colitis. *EBioMedicine*10.1016/j.ebiom.2019.10.002 (2019). 10.1016/j.ebiom.2019.10.002 PMC6838378 31628021

[61] Brooks SPJ, McAllister M, Sandoz M, Kalmokoff ML (2003). Culture-independent phylogenetic analysis of the faecal flora of the rat. Can. J. Microbiol..

[62] Manichanh C (2010). Reshaping the gut microbiome with bacterial transplantation and antibiotic intake. Genome Res..

[63] Pellizzon, M. A. & Ricci, M. R. Choice of laboratory rodent diet may confound data interpretation and reproducibility. *Curr. Dev. Nutr.*10.1093/cdn/nzaa031 (2020). 10.1093/cdn/nzaa031 PMC7103427 32258990

[64] Pellizzon MA, Ricci MR (2018). The common use of improper control diets in diet-induced metabolic disease research confounds data interpretation: the fiber factor. Nutr. Metab..

[65] Čoklo M, Maslov DR, Kraljević Pavelić S (2020). Modulation of gut microbiota in healthy rats after exposure to nutritional supplements. Gut Microbes.

[66] Le Bras A (2022). Reducing cage effects in mouse microbiome studies. Lab Anim..

[67] Carbone L (2021). Estimating mouse and rat use in American laboratories by extrapolation from Animal Welfare Act-regulated species. Sci. Rep..

[68] Schweinfurth MK (2020). The social life of Norway rats (*Rattus norvegicus*). eLife.

[69] Makowska IJ, Weary DM (2020). A good life for laboratory rodents?. ILAR J..

[70] McMurdie PJ, Holmes S (2013). phyloseq: an R package for reproducible interactive analysis and graphics of microbiome census data. PLoS ONE.

[71] Dixon P (2003). VEGAN, a package of R functions for community ecology. J. Veg. Sci..

[72] Adler D, Nenadic O, Zucchini W (2003). Rgl: a r-library for 3d visualization with opengl. Proc. 35th Symposium of the Interface: Computing Science and Statistics.

[73] Carmona-Saez P, Pascual-Marqui RD, Tirado F, Carazo JM, Pascual-Montano A (2006). Biclustering of gene expression data by non-smooth non-negative matrix factorization. BMC Bioinformatics.

[74] Kolde, R. pheatmap: pretty heatmaps *CRAN* https://CRAN.R-project.org/package=pheatmap (2015).

[75] Durinck S, Spellman PT, Birney E, Huber W (2009). Mapping identifiers for the integration of genomic datasets with the R/Bioconductor package biomaRt. Nat. Protoc..

[76] Kuleshov MV (2016). Enrichr: a comprehensive gene set enrichment analysis web server 2016 update. Nucleic Acids Res..

