# Supplementary material for: Rat microbial biogeography and age-dependent lactic acid bacteria in healthy lungs
Source: Lab Anim (NY). 2024 Jan 31;53(2):43–55. doi: 10.1038/s41684-023-01322-x (PMC10834367; doi:10.1038/s41684-023-01322-x)
Supplement: Supplementary file 1 — Supplementary Figs. 1–5. [file 41684_2023_1322_MOESM1_ESM.pdf]

lab animal

Article

https://doi.org/10.1038/s41684-023-01322-x

Rat microbial biogeography and age-dependent lactic acid bacteria in healthy lungs

In the format provided by the authors and unedited

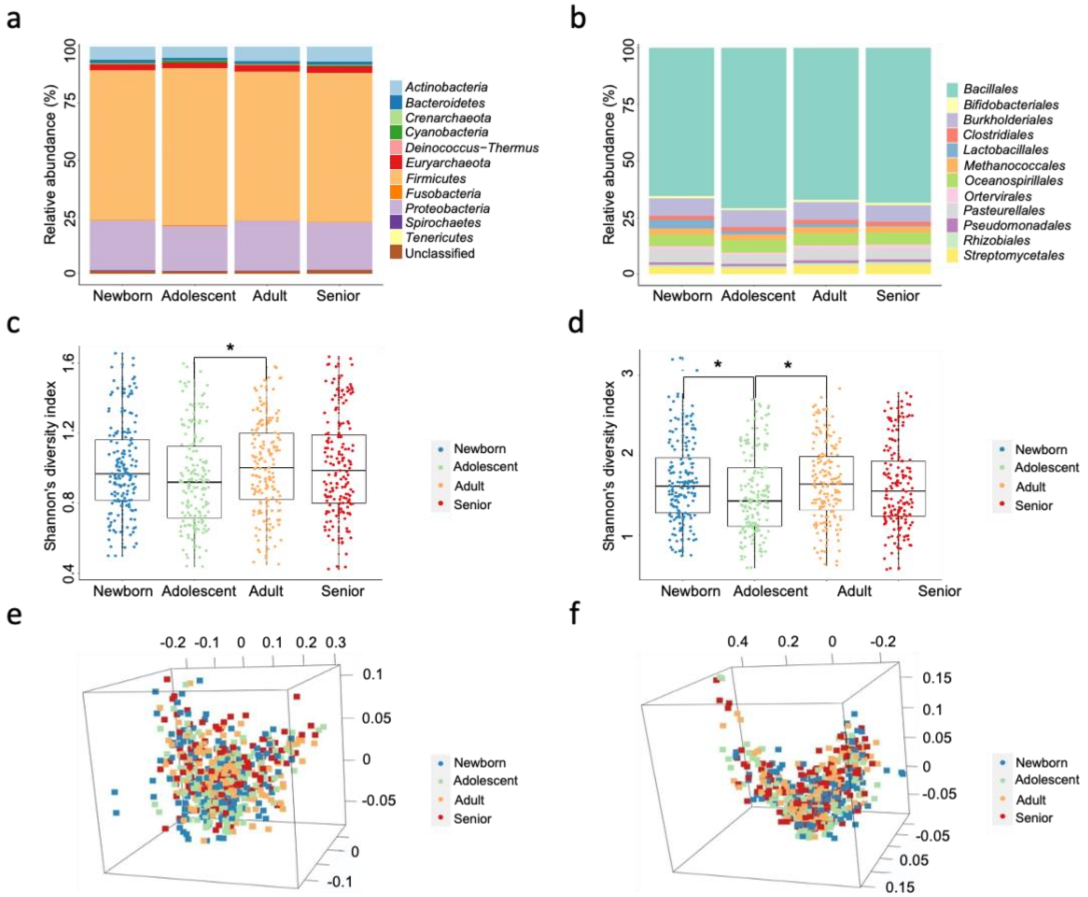

Figure S1. Microbial composition and diversity comparisons of the four developmental stages in F344 rats at the phylum and the order levels. a-b, Taxa composition bar plots illustrate the microbial relative abundance (%; in y-axis) of the top 12 most abundant taxa of the four developmental stages at the phylum (**a**) and the order level (**b**). **c-d,** Shannon's diversity index (in y-axis) of the four stages at the phylum (**c**) and the order level (**d**). Pairwise Wilcoxon rank sum tests with BH correction were used to test for diversity differences between stages. Only statistically significant comparisons (adjusted $P < 0.05$) are marked with a single star (*), and adjusted *P*-values < 0.01 are marked with two stars (**). **e-f,** 3D-view PCoA of Bray-Curtis dissimilarity between samples colored in four different colors according to each developmental stage at the phylum (e) and the order level (f).

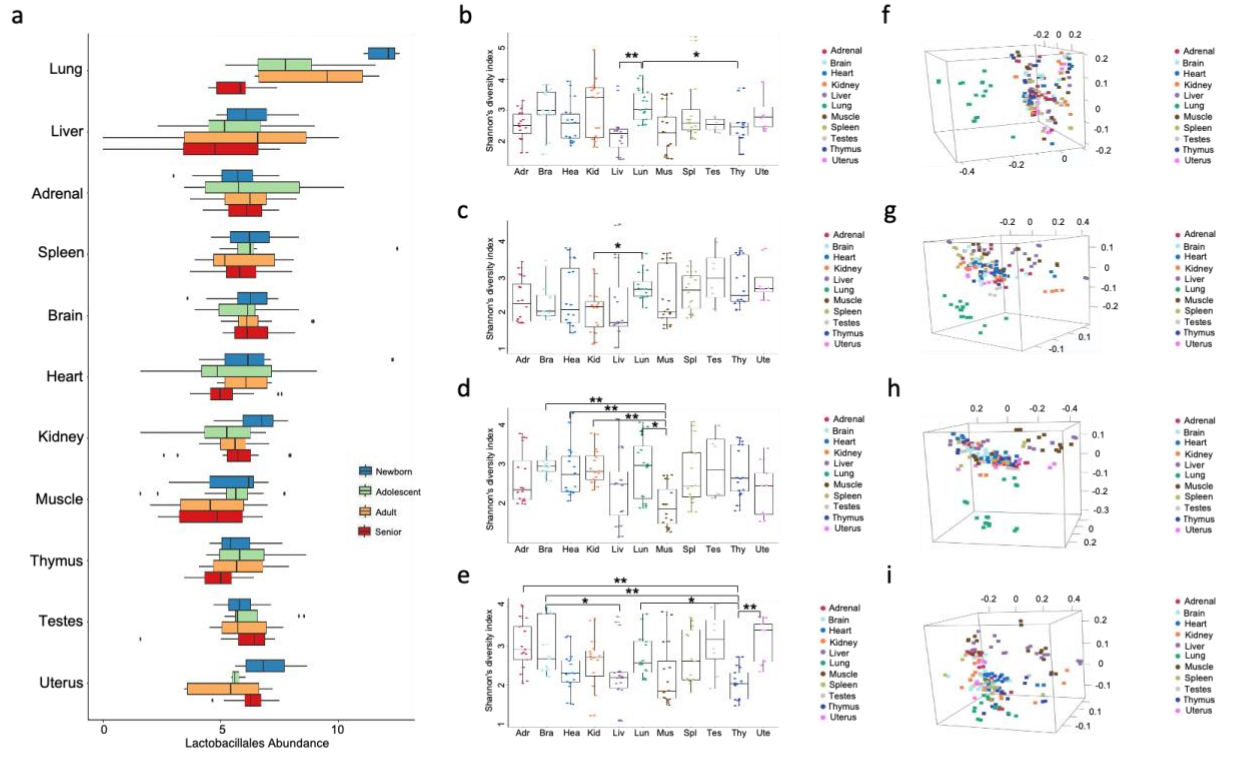

Figure S2. Tissue-level microbial composition and diversity comparisons during the four developmental stages. a, Lactobacillales abundance in the 11 tissues and the four developmental stages. **b-e,** Species-level Shannon's diversity index (in y-axis) of the 11 tissues at the newborn (**b**; n=168), adolescent (**c**; n=162), adult (**d**; n=163), and senior stage (**e**; n=167). Pairwise Wilcoxon rank sum tests with BH correction were used to test for diversity differences between tissues. Only statistically significant comparisons ($P < 0.05$) are marked with a single star (*), and *P*-values < 0.01 are marked with two stars (**). **f-i,** 3D-view species-level PCoA of Bray-Curtis dissimilarity between samples colored in 11 different colors according to each tissue type at the newborn (**f**), adolescent (**g**), adult (**h**), and senior stage (**i**).

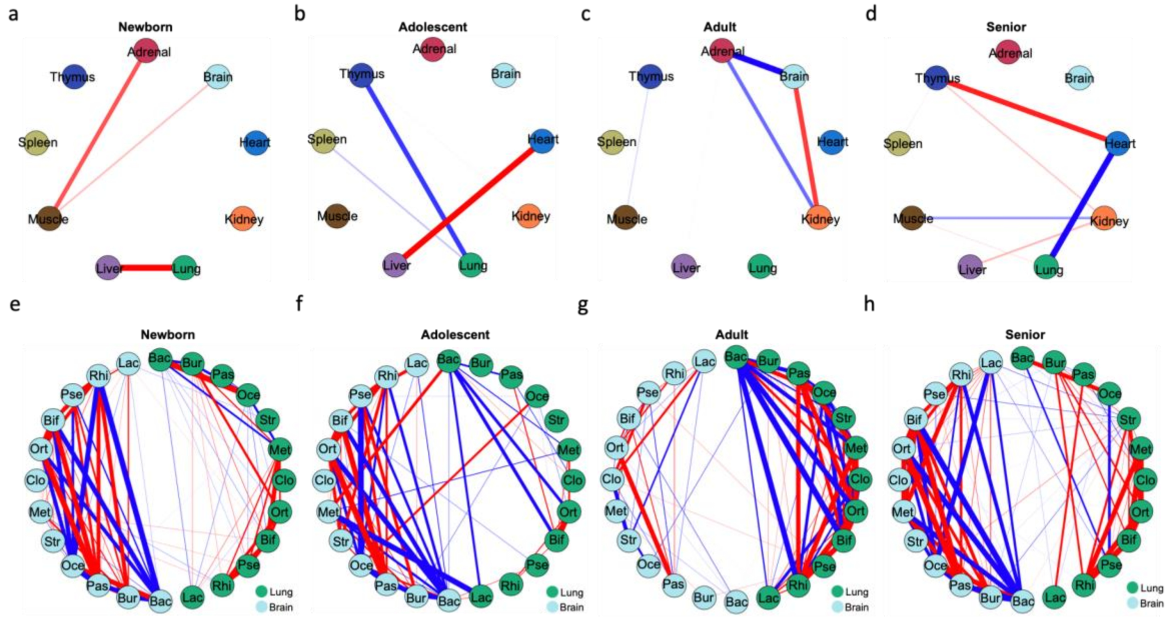

Figure S3. *Lactobacillus* abundance and the lung–brain axis during the four developmental stages. a-d, Circular correlation networks display the Spearman's correlations for the *Lactobacillus* abundance in each tissue type at the newborn (**a**; n=168), adolescent (**b**; n=162), adult (**c**; n=163), and senior stage (**d**; n=167). **e-h,** Circular correlation networks display the Spearman's correlations for the top 12 most abundant orders of association between lungs (green) and brain (light blue) at the newborn (**e**; n=168), adolescent (**f**; n=162), adult (**g**; n=163), and senior stage (**h**; n=167). The strength of the correlations are proportional to their absolute correlation coefficients, and the color indicate the direction of the correlations (red for positive, and blue for negative correlations). Only strong correlations with absolute correlation coefficients greater than 0.5 were shown.

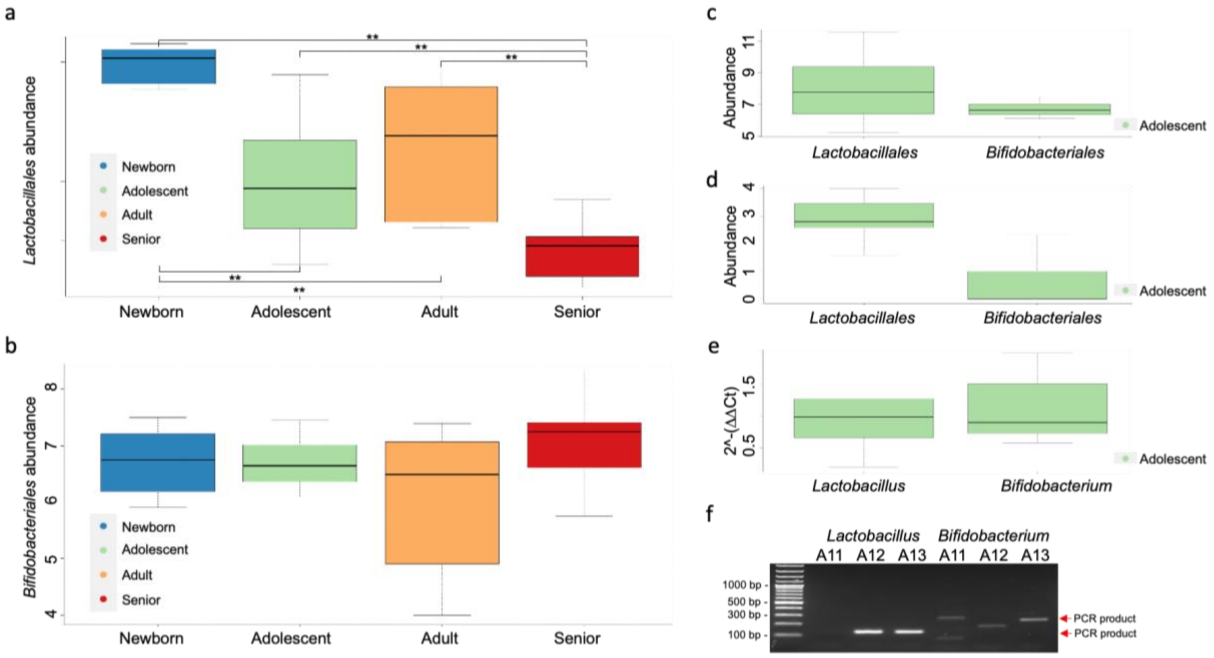

Figure S4. Boxplots and agarose gel showing the abundance of Lactobacillales and Bifidobacteriales in lungs of both the discovery and validation datasets.

a,. Boxplot illustrates the microbial abundance of Lactobacillales (in y-axis) at the newborn (n=168), adolescent (n=162), adult (n=163), and senior (n=167) stages in the discovery dataset. **b,** Boxplot illustrates the microbial abundances of Bifidobacteriales (in y-axis) at the newborn (n=168), adolescent (n=162), adult (n=163), and senior (n=167) stages in the discovery dataset. **c-d,** Boxplots illustrate the microbial abundances of Lactobacillales and Bifidobacteriales (in y-axis) in the discovery (**c**; n=162+162) and validation dataset 1 (**d**; n=6+6), which were based on RNA-Seq-derived adolescent rats' microbial results. Abundance in (**a-d**) were log2 (species counts+1). **e,** Boxplot illustrates the RT-PCR microbial abundance (2^-(ΔΔCt)) in a total of 15 and 30 *Lactobacillus and Bifidobacterium* spp. (in y-axis; n=6+6), respectively, from the validation dataset 1. Pairwise Wilcoxon rank sum tests with BH correction were used to test for diversity differences between groups for boxplots in (**a-e**). Only statistically significant comparisons ($P < 0.05$) are marked with a single star (*), and P-values < 0.01are marked with two stars (**). **f,** Agarose gel (2%) electrophoresis of the amplified 126 bp *Lactobacillus* spp. and 100-244 bp *Bifidobacterium* spp. PCR products, respectively, in the three subjects from the validation dataset 2.

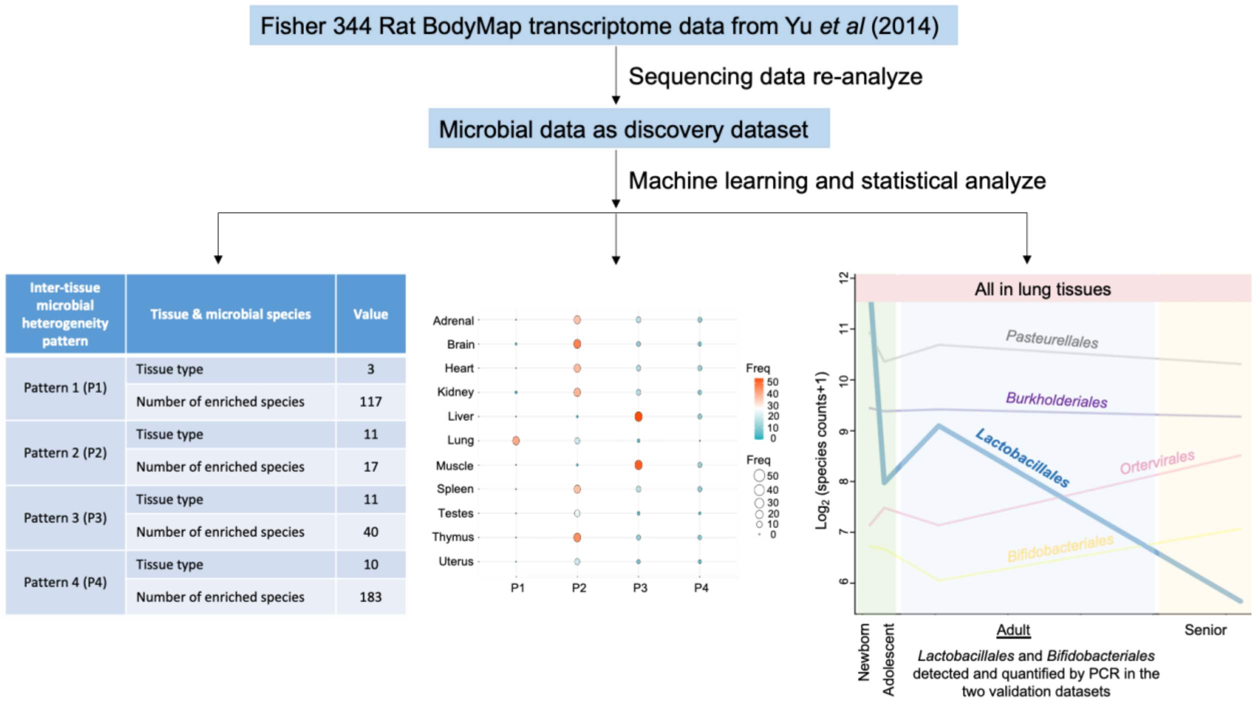

Figure S5. The general workflow for the study.
